# Autophagy impairment in human bile duct carcinoma cells

**DOI:** 10.3389/fphys.2023.1249264

**Published:** 2023-09-29

**Authors:** Simonetta Petrungaro, Valerio de Franchis, Antonio Filippini, Antonio Facchiano, Eugenio Gaudio, Claudia Giampietri

**Affiliations:** ^1^ Department of Anatomy, Histology, Forensic Medicine and Orthopedics, Sapienza University of Rome, Rome, Italy; ^2^ Laboratory of Molecular Oncology, Istituto Dermopatico dell'Immacolata (IDI-IRCCS), Rome, Italy

**Keywords:** autophagic genes and proteins, cholangiocytes, proliferation markers, caspase, cancer

## Abstract

Bile duct epithelial cells, named cholangiocytes, may undergo a neoplastic transformation leading to cholangiocarcinoma. The role autophagy plays in cancer is still debated and few information are available in cholangiocarcinoma. We report *in vitro* data, at least in part validated *in vivo,*i ndicating that autophagy is impaired in intrahepatic cholangiocarcinoma cells, as compared to healthy cholangiocytes, evaluated through LC3II and p62 Western blot analyses. Autophagy impairment was found to be associated with low expression of TFEB protein and high expression of three proteins i.e., c-FLIP, caspase-10 and cleaved BCLAF-1, as compared to healthy cholangiocytes. We highlight biological effects of autophagy impairment in cholangiocarcinoma showing that autophagy induction, via rapamycin, as well as caspase inhibition, via Q-VD-OPh, are able to reduce proliferation marker PCNA level, colony size and protein content of cultured cholangiocarcinoma cells. The increased protein expression of p62, c-FLIP, caspase-10 observed *in vitro* in cholangiocarcinoma cells was paralleled by significant increase at gene expression levels *in vivo*; in fact, significant increase of transcript levels of p62, c-FLIP and caspase-10 was observed in 34 biopsies from human cholangiocarcinoma patients compared to 9 biopsies from 9 healthy controls, as reported in the GEPIA2 public database. The significant increase of p62 level in cholangiocarcinoma was found as a relatively uncommon finding in solid cancers, since it was also found in only 7 cancer types out of 31 cancer types investigated, including melanoma and hepatocarcinoma. In conclusion, we present data suggesting a molecular machinery controlling autophagy in cholangiocytes and autophagy impairment in cholangiocarcinoma.

## 1 Introduction

Cholangiocarcinoma (CCA) is a rare and highly lethal malignant liver tumour arising from bile duct epithelial cells (cholangiocytes) transformation. It is classified into intrahepatic cholangiocarcinoma (iCCA) and extrahepatic cholangiocarcinoma (eCCA), depending on its localization (Kendall et al., 2019). eCCA includes perihilar (pCCA) and distal (dCCA) cholangiocarcinoma.

In the last decades autophagy deregulation has been shown to be reported in CCA (Sasaki et al., 2015). Autophagy is a highly conserved catabolic mechanism operating at basal levels in cells. Through the degradation and recycling of macromolecules and cellular components, autophagy mediates quality-control and homeostasis. The phosphatidylethanolamine (PE) conjugated LC3 is a protein known as LC3-II, its levels correlate with autophagosomes number and it is commonly used as a marker of autophagosomes. The protein itself, as it localizes on the autophagosomes, is degraded following autophagosomes and lysosomes fusion (Mizushima, 2018). Another protein commonly analysed to monitor autophagy is p62, which possesses a short LC3 interaction region causing p62 to be specifically degraded by autophagy. Since p62 degradation is dependent on autophagy, the level of p62 increases in response to autophagy inhibition (Bjorkoy et al., 2009; Klionsky et al., 2021).

The role of autophagy in cancer appears controversial since it acts as a tumour suppressor or a tumour promoter, depending on cancer types. For instance, in melanoma, autophagy is reported to be either as tumour-suppressive in early stages and tumour-promoting in later stages (Ndoye and Weeraratna, 2016). Autophagy-related genes are frequently deleted, silenced, or mutated in different human tumours, thus supporting the autophagy tumour-suppressing properties. It has been also reported that inhibiting autophagy may activate proliferation pathways (Manning and Toker, 2017). While during cancer initiation autophagy may suppress tumour progression and autophagy deregulation may contribute to genomic instability, in the late cancer stages autophagy may facilitate tumour progression allowing cancer cell survival, particularly in the presence of therapy-induced stress (Mancinelli et al., 2017; Assi and Kimmelman, 2023). Finally, autophagy may inhibit cell growth at least in part by promoting protein and/or organelle turnover (Wang and Levine, 2010).

Several experimental data suggest a deregulated autophagy in CCA at the initial steps of cholangio carcinogenesis. In fact, precursor biliary intraepithelial neoplasia lesions showed higher levels of LC3-II and p62 compared to normal biliary ducts, indicating uncomplete autophagic process, reinforcing the hypothesis that autophagy inhibition may promote carcinogenic transformation (Perez-Montoyo, 2020). Recently, germline mutation of ATG7 (a protein playing an essential role during autophagosome formation) has been shown to be associated to increased p62 levels in bile duct epithelial cells and to familial CCA (Greer et al., 2022). Furthermore, additional genetic events occurring in iCCA, such as *KRAS* or *BRAF* mutations, may also indirectly activate mTOR and this may lead to autophagy inhibition. Agents able to modulate autophagy at different steps might be combined with chemotherapy or radiotherapy in order to increase the beneficial effects of a single approach. Interestingly, in iCCA mTOR inhibitors, by stimulating autophagy, may be a therapeutic promising approach (Koustas et al., 2021; Lu et al., 2021).

Recently, autophagy activity level in CCA samples derived from different anatomical locations has been investigated. Results indicated an inhibited autophagy in iCCA and pCCA tumour tissues. Conversely, activated autophagy was found in dCCA, particularly in samples displaying low Ki67 index (Lendvai et al., 2021). CCA is a highly heterogeneous tumour and such feature may be associated to differences in autophagy. Therefore, further details elucidating autophagy control may highlight new possible therapeutic approaches to therapy (Koustas et al., 2021).

In the present study we investigated autophagy in a iCCA cell line named HuCCT1 (Yoshikawa et al., 2009) *vs* healthy human cholangiocyte cells (H69), in order to clarify mechanisms underlying autophagy control in *in vitro* conditions.

## 2 Materials and methods

### 2.1 Cells culture and reagents

HuCC-T1 and OZ bile duct carcinoma cell lines were purchased from Tebu-bio (Magenta, MI, Italy). OZ were previously established by Prof. Seishi Nagamori (Department of Virology II, National Institute of Infectious Diseases, 1-23-1 Toyama, Shinjyuku-ku, Tokyo 162-8640 Japan).

H69 immortalized normal human cholangiocyte cells were kindly provided by Prof. Vincenzo Cardinale (Sapienza University of Rome, Italy). H69 cells show phenotypic characteristics of normal adult cholangiocytes, are non-tumorigenic when injected into athymic nude mice and do not express alpha-fetoprotein (Pfeifer et al., 1993). Cells were cultured in DMEM (Gibco-Invitrogen, Carlsbad, CA, USA) containing high glucose enriched with 10% fetal bovine serum, glutamine (2 mmol/l), in presence of penicillin (100 U/ml) and streptomycin (100 μg/ml). Cells were maintained at 37°C in a humidified 5% CO_2_ atmosphere.

Bafilomycin A1 was purchased from Sigma-Aldrich (Milano, Italy) and was used at 100 nM during the last 3 h treatment. Rapamycin was purchased from Sigma and used 54 nM for 48 h according to our previous studies (Giampietri et al., 2012). EBSS was purchased from Sigma-Aldrich and used to carry out the 3-h starvation experiments.

The caspase-10 inhibitor Q-VD-OPh was purchased from Selleckchem (Houston, 77014 USA) and used 25 μM for 48 h.

### 2.2 Nuclear and cytoplasmic fractionation

Briefly, from confluent dishes, cells were harvested and pelleted in centrifuge for 4 min at 1000 rpm. Pellet was washed with PBS and cell suspension was centrifuged again. Then hypotonic buffer (Hepes 10 mM pH7.9, EDTA 1 mM, KCl 60 mM, DTT 1 mM, PMSF 1mM, containing protease inhibitor cocktail (Sigma-Aldrich) and 0.1% NP-40) was added on ice. The solution was centrifuged at 3000 rpm for 15 min at 4°C to separate nuclei from supernatant, corresponding to cytoplasmic fraction used in Western blot experiments. Then nuclei were resuspended and centrifuged over sucrose cushion (60% in H_2_O). The nuclear pellet was suspended in lysis buffer (Tris-HCl 200 mM pH7.8, KCl 60 mM, DTT 1 mM, PMSF 1 mM, containing protease inhibitor cocktail) performing freeze-thaw cycles in order to lyse nuclear membranes.

After a final centrifugation, supernatant, corresponding to nuclear fraction, was used in Western blot experiments (D’Arcangelo et al., 2019).

### 2.3 Western blotting

Cells were washed two times with pre-chilled PBS (Phosphate Buffered Saline) purchased from Sigma-Aldrich and lysed. Lysis Buffer 10x (Cell Signalling, Danvers, MA, USA) was diluted in the presence of 2% SDS (Sodium Dodecyl Sulphate) and proteases’ inhibitors (Sigma-Aldrich). Lysates were also sonicated through a sonicator (Branson, Danbury, USA) for 10 s at 50% amplitude. Total cell lysates were incubated for 10min on ice, then sonicated and centrifuged at 4°C for 15 min at 14.000 g to remove cell debris.

Protein concentration was determined by micro BCA assay (Pierce, Thermo Scientific, Rockford, IL, USA) and samples were boiled at 95°C for 5 min following Laemmli Buffer addition (0,04% Bromophenol blue, 40% Glycerol, 2% SDS, 20% ß-mercaptoethanol, 250 mM Tris HCl pH.6.8, all purchased from Sigma-Aldrich) (Giampietri et al., 2006).

Proteins were separated by SDS–PAGE and transferred on Polyvinylidene fluoride (PVDF) or Nitrocellulose membranes (Amersham Bioscience, Piscataway, NJ, USA). Membranes were probed using the following antibodies: anti-β-Actin-HRP (Sigma-Aldrich A3854); anti-LC3 (Cell Signalling 2775); anti-caspase-10 (Santa Cruz SC-7955); anti-c-Flip (Cell Signalling D5J1E mAb 56343); anti-TFEB (Bethyl laboratories A303-673A-M); anti p62 (Abcam) ab56416); anti-PCNA (Abcam ab2426); anti-BCLAF (also called BTP; Bethyl laboratoriesA300-608A-T); anti-PARP (cell signalling 9542S); anti-GAPDH (Sigma-Aldrich G8795).

For LC3 we particularly focused on LC3-II. More in detail, LC3-I is conjugated to phosphatidylethanolamine (PE) for the generation of LC3-II form. The lipophilic character of PE group allows the insertion of LC3-II into the membranes of autophagosomes making LC3-II a marker of autophagosomes. Subsequently, LC3-II protein is degraded when autophagosomes fuse with lysosomes. Since LC3-II turnover may be very fast, we used bafilomycin A1, an inhibitor of autophagosomes and lysosomes fusion, as already described, to block LC3-II degradation into autophagosomes in order to monitor autophagosome formation (Klionsky et al., 2021).

Secondary antibodies were horseradish peroxidase-conjugated anti-mouse or anti-rabbit (Bio-Rad, Hercules, CA, USA). Membranes were washed with Tris-buffered saline (Medicago, Uppsala, Sweden) with 0.1% Tween-20 (Sigma-Aldrich) and developed through the chemiluminescence system (Amersham Bioscience) using the ChemiDoc image analyser, Image lab software for densitometric quantifications (Bio-Rad, Hercules, CA, USA). Quantification of the reported blots was performed in triplicate.

### 2.4 Immunofluorescence

20.000 HuCC-T1 or H69 cells were seeded onto glass coverslips precoated with poly-D-lysine 5 ug/ml in 12-well dishes and allowed to attach overnight. After one PBS wash, cells were fixed with 4% PFA for 20 min. After 3 washes in PBS (5 min each), coverslips were incubated with 10% Normal Donkey Serum (NDS); 0,1% Triton X-100; 0,1M glycine in PBS (blocking solution) 1 h at Room Temperature (RT). Coverslips were then incubated with primary anti-TFEB antibody diluted 1:100 in blocking solution for 1 h at RT. After three 5 min washes in PBS, coverslips were incubated with secondary antibody Cy3, anti-rabbit IgG, diluted 1:400 in PBS for 1 h at RT. After three 5 min washes in PBS, coverslips were mounted with Vectashield (antifade mounting medium containing DAPI for nucleic acid staining; Vector laboratories, DBA, Milan; Italy) onto microscope slides. Photographs were acquired using an Axio imager A2 system equipped with an AxiocamHRc, with Axiovision Release 4.8.2 software (Zeiss, Oberkochem, Germany).

### 2.5 Investigating gene expression levels in public databases

p62, c-FLIP and Caspase-10 gene expression levels were investigated on TCGA datasets available on the GEPIA2 portal (at http://gepia2.cancer-pku.cn/#index) (Tang et al., 2019).

This portal reports expression data for 31 cancer types in a total of about 15 thousand patients and healthy controls. Cholangiocarcinoma data investigated in the present study were derived from 34 cholangiocarcinoma (CCA) biopsies and 9 healthy control biopsies, as filtered by the “subtype filter” option.

### 2.6 Statistical analyses

Data were expressed as the mean ± Standard Error of the mean and statistical analysis was carried out by One-way ANOVA. Differences were considered significant for *p* ≤ 0.05.

Statistics for gene expression data were from the GEPIA2 portal according to the methods reported in http://gepia2.cancer-pku.cn/#analysis. The method for differential analysis is one-way ANOVA, using disease state (Tumor or Normal i.e., Ctrl) as variable for calculating differential expression, according to methods reported in http://gepia2.cancer-pku.cn/#analysis. The significance threshold was set at *p* ≤ 0.01.

## 3 Results

### 3.1 Autophagy impairment in iCCA *in vitro* models

To investigate differences between CCA cells and cholangiocytes we analysed two well-known autophagy markers, LC3 and p62 (Klionsky et al., 2021), in HuCCT1 (iCCA cells) and H69 (cholangiocytes).

**FIGURE 1:**
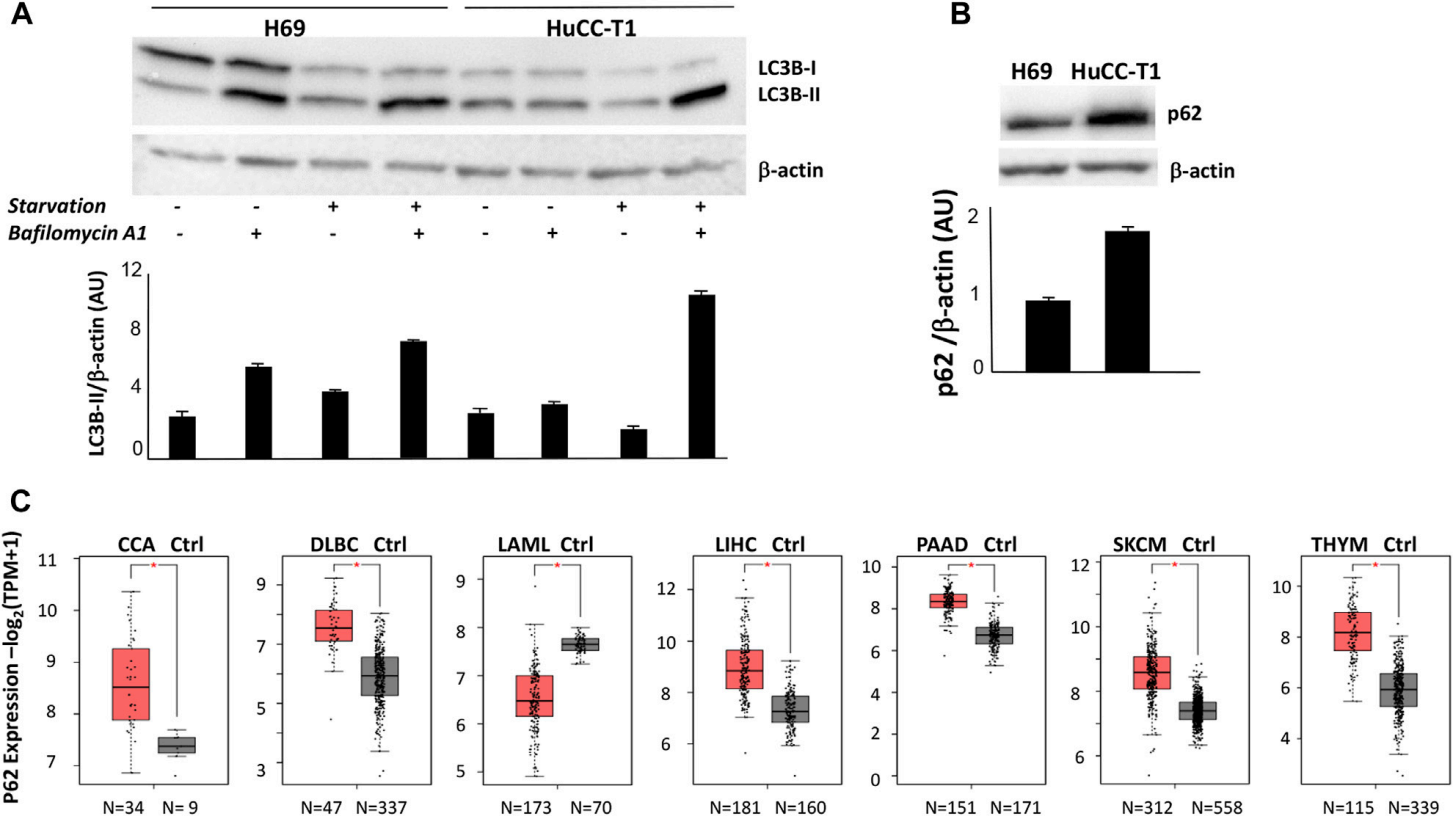
**(A)** Representative Western blot experiment showing strong LC3-II signal in H69 but not HuCC-T1 in the presence of bafilomycin under standard condition. Upon starvation both H69 and HuCCT1 display strong LC3II signal. β-Actin was used as loading control. Blot is representative of three independent experiments. Quantification of the reported blot is shown, performed in triplicate. **(B)** Representative Western blot experiment shows strong p62 in HuCCT1 as compared to H69 cells. β-Actin was used as loading control. Blot is representative of three independent experiments. Quantification of the reported blot is shown, performed in triplicate. **(C)** p62 expression was investigated and was found significantly increased in 7 out of 31 cancer types, according to TCGA data reported in GEPIA2 platform. The box plot of the cancer type is reported, obtained by clicking «all subtypes» form the «filter subtype» option. CCA, Cholangiocarcinoma; DLBC, Lymphoid Neoplasm Diffuse Large B-cell Lymphoma; LAML, Acute Myeloid Leukemia; LIHC, Liver hepatocellular carcinoma; PAAD, Pancreatic adenocarcinoma; SKCM, Skin Cutaneous Melanoma; THYM, Thymoma.

As shown in Figure 1A, autophagy is active in H69 cells; in fact, LC3-II accumulation is found in full medium culture in the presence of bafilomycin A1 lane. Under the same conditions, HuCCT-1 cells show low level of autophagy. Autophagy was then induced through starvation, by replacing full medium with Earle’s balanced salt solution (EBBS) (Klionsky et al., 2021). Under such conditions, LC3-II accumulate in bafilomycin A1 lane, in both HuCCT1 and H69 cells. We therefore concluded that autophagosome formation is higher in H69 cells as compared to HuCCT1 cells under basal culture conditions, and autophagosome formation is induced in HuCCT1 cells upon starvation suggesting that in iCCA cells nutrient deprivation elicits an acute autophagic response.

We then evaluated p62 protein expression by Western blot analyses and found higher level in HuCCT-1 cells as compared to the control, under culture in full medium, as shown in Figure 1B, thus confirming an impaired autophagy in iCCA cells. This result reinforces in our experimental model previous data obtained by immunohistochemistry on human iCCA sections (Chen et al., 2023). P62 mRNA expression level was then evaluated in CCA biopsies, by interrogating public databases available on GEPIA2 portal (Figure 1C). A significant increase of p62 transcripts levels was found in CCA biopsies as compared to normal healthy controls (34 *vs.* 9 samples). The significant increase of p62 gene expression levels appears as a relatively uncommon feature, since it was found in only 7 out of 31 cancer types, including 5 solid cancers: cholangiocarcinoma, liver hepatocellular carcinoma, pancreatic adenocarcinoma, melanoma and thymoma (Figure 1C).

### 3.2 TFEB protein expression and subcellular localization is altered in iCCA cells

We evaluated possible mechanisms underlying autophagy impairment in HuCC-T1. We first focused on TFEB, a transcription factor controlling both cholangiocytes differentiation (Pastore et al., 2020) and cell autophagy promotion (Di Malta et al., 2019).

**FIGURE 2:**
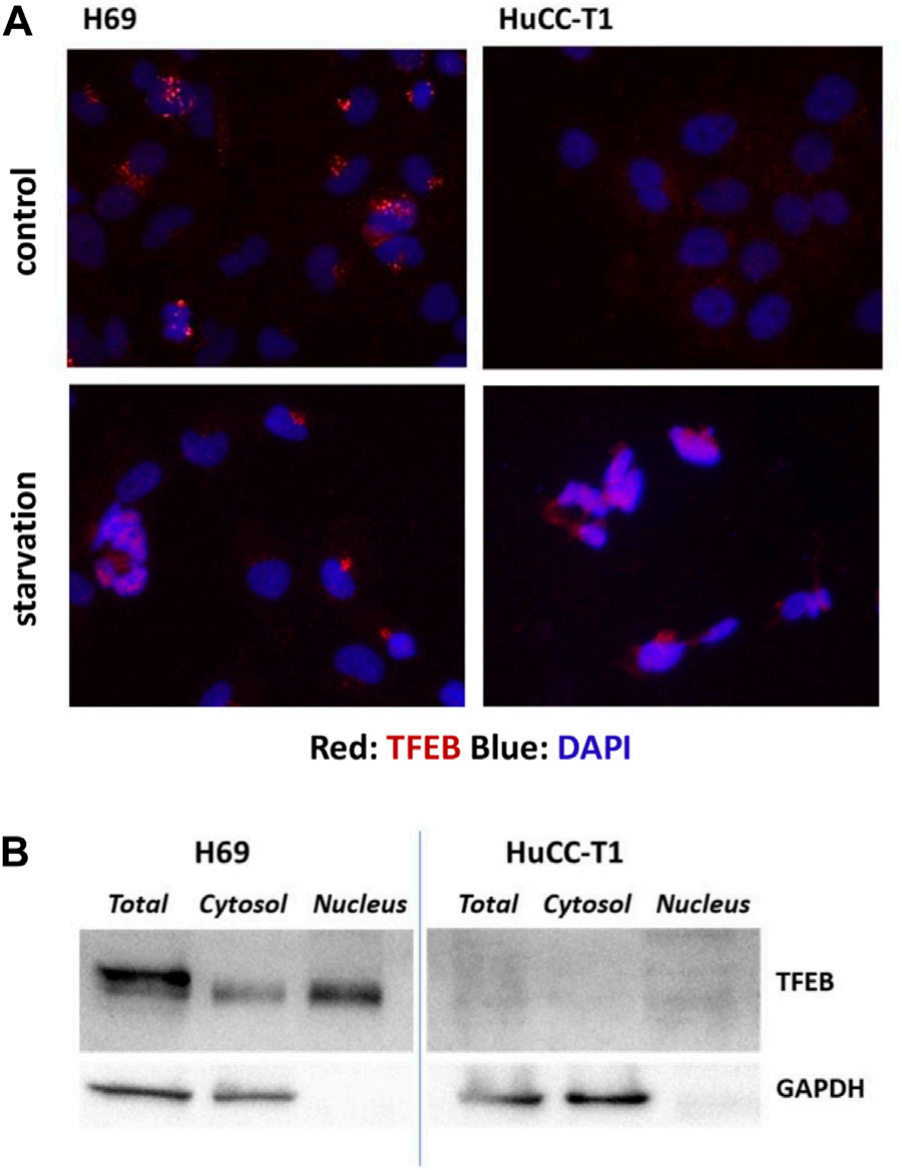
**(A)** Representative immunofluorescence showing immunostaining (red) of TFEB in H69 and HuCC-T1 cells under control and starvation condition. Nuclei were stained with DAPI (blue). Magnification ×400. **(B)** Representative Western blot experiment showing TFEB presence in total lysate, cytosol and nucleus samples of H69 cells while almost TFEB absence in samples from HuCCT1 cells (either nucleus or cytosol). GAPDH was used as loading control for cytosol and total lysate samples. GAPDH absence in nucleus samples indicates their purity. Blots are representative of three independent experiments.

TFEB shuttles from the cytosol to the nucleus depending on the cellular condition and on its phosphorylation by mTORC complex 1 (mTORC1). When TFEB localizes to cytosol it is inactive, while when it is localized in the nucleus activates specific target genes, including autophagic genes. Immunofluorescence staining of TFEB in H69 and HuCC-T1 cells shows TFEB localization in nuclear and perinuclear regions of H69 cells while only minimal diffuse cell staining in HuCC-T1 under control conditions. Upon starvation, nuclear TFEB localization is evident also in HuCC-T1 cells (Figure 2A). Such result is consistent with LC3 data shown in Figure 1A, indicating that nutrient deprivation elicits an acute autophagic response also in HuCC-T1 cells.

To further investigate TFEB protein expression in the two cells types, Western blotting analyses were carried out on nucleus, cytosol and total cell lysates of both H69 and HuCCT1 cells in control conditions (Figure 2B). TFEB was confirmed to be almost absent in HuCCT1 cells (either nucleus or cytosol) and present in controls (both nucleus and cytosol).

### 3.3 c-FLIP, caspase-10 and BCLAF-1 proteins are differently expressed in iCCA cells

**FIGURE 3:**
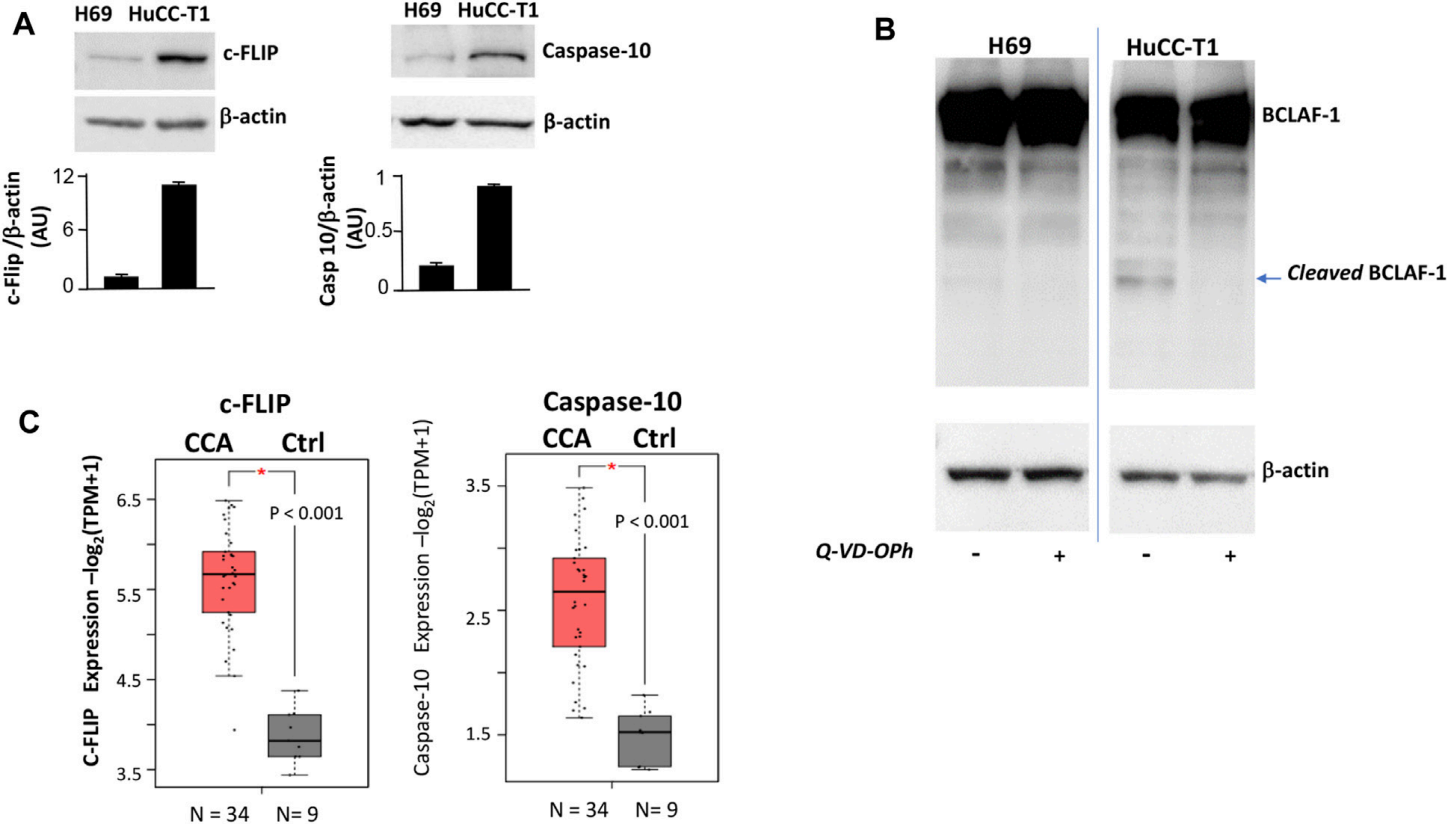
**(A)** Western blot experiments showing strong c-FLIP and caspase-10 bands in HuCC-T1 but not in H69 cells. β-Actin was used as loading control. Blots are representative of three independent experiments. Quantification of the reported blot is shown, performed in triplicate. **(B)** Representative Western blot experiment showing full length BCLAF-1 in both H69 and HuCC-T1 while cleaved BCLAF-1 only in HuCC-T1 in the absence of Q-VD-OPh (caspase-10 inhibitor). Blots are representative of three independent experiments. β-Actin was used as loading control. **(C)** c-FLIP and caspase-10 transcripts expression levels were investigated and were found significantly increased in CCA patients, according to TCGA data reported in GEPIA2 platform. * indicates *p* ≤ 0.01.

As an additional characterization, the expression of three proteins (c-FLIP, caspase-10 and BCLAF-1) was investigated, since such proteins were previously shown to be directly involved in autophagy control (Lamy et al., 2013). Higher expression of both c-FLIP (55 KDa) and caspase-10 was observed in HuCC-T1 as compared to H69 cells (Figure 3A), suggesting possible cleavage enzymatic activity of c-FLIP-caspase-10 complex.

To address such hypothesis, BCLAF-1 protein, a known target of c-FLIP-caspase-10 complex (Lamy et al., 2013), was investigated in Western blotting analyses (Figure 3B). The cleaved form of BCLAF-1 was found in HuCC-T1 but not in H69, and this form disappeared when cells were treated with the caspase inhibitor Q-VD-OPh. By interrogating public databases available on GEPIA2 portal, c-FLIP and caspase-10 transcripts levels were also analyzed and found higher in CCA biopsies, as compared to healthy controls (Figure 3C).

We then hypothesize that BCLAF-1 cleaved form may be responsible, at least in part, for the impaired autophagy found in iCCA cells, as better discussed in the Discussion section.

To test our hypothesis regarding a key role of c-FLIP-caspase10 complex enzymatic activity in autophagy impairment, we investigated the effects of the caspase inhibitor Q-VD-OPh on LC3-II in HuCC-T1 cells. As shown in Supplementary Figure S1, LC3-II accumulation was increased in HuCCT1 cells upon Q-VD-OPh treatment in the presence of bafilomycin A1, as compared to untreated cells by Western blot experiments. We therefore concluded that pharmacological caspase inhibition may lead, at least in part, to autophagy activation in HuCCT1 cells.

### 3.4 Autophagy activation is associated with reduced proliferation marker and unchanged apoptosis marker in iCCA cells

**FIGURE 4:**
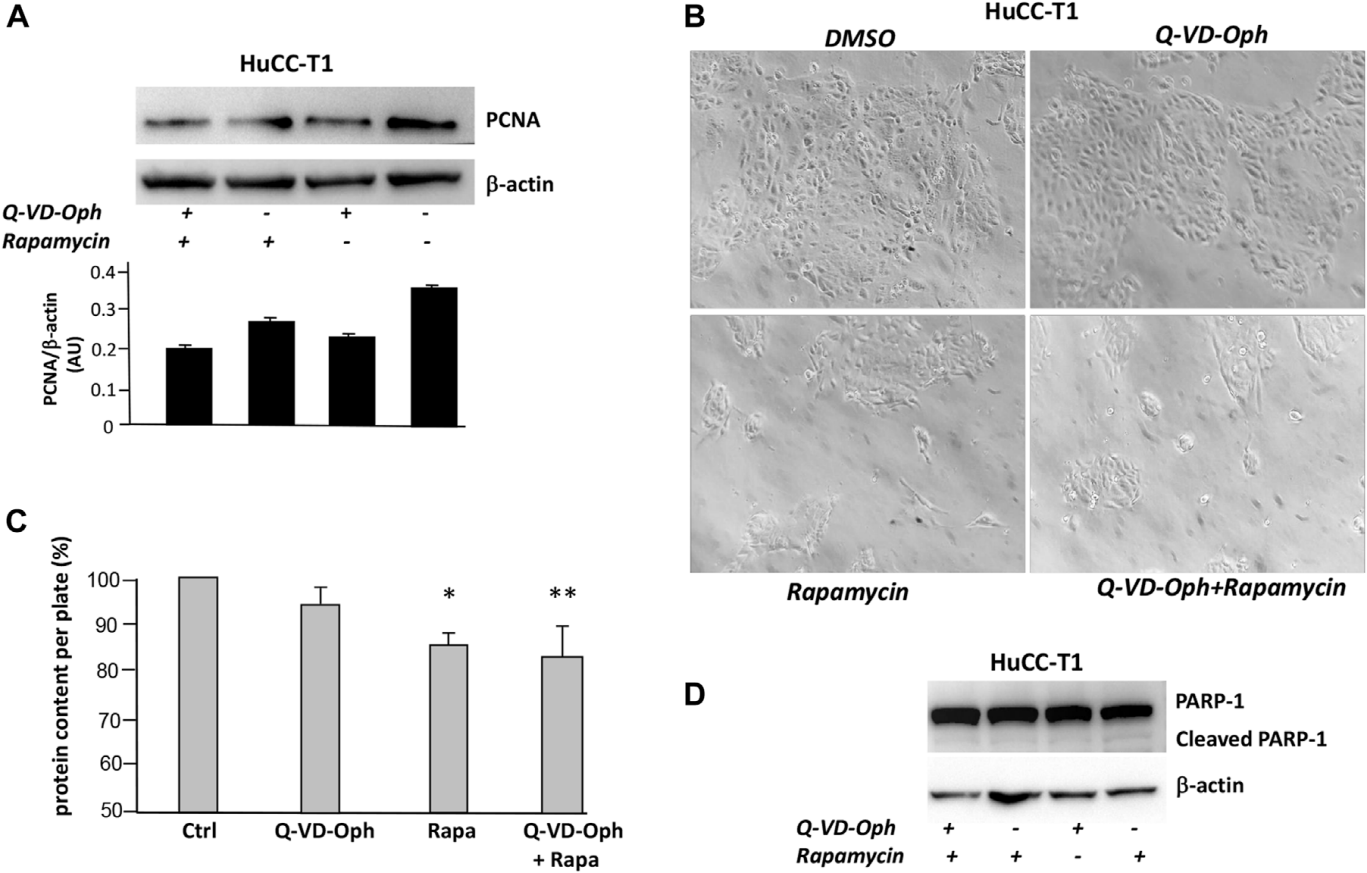
**(A)** Representative Western blot experiment showing the effect of Q-VD-OPh (caspase-10 inhibitor), rapamycin and their combination in reducing the level of PCNA (proliferation marker) in HuCC-T1 cells. β-Actin was used as loading control. Blot is representative of three independent experiments. Quantification of the reported blot is shown, performed in triplicate. **(B)** Phase contrast representative images of cultured HuCC-T1 showing the effect of Q-VD-OPh (caspase-10 inhibitor), rapamycin and their combined treatment in affecting cell population on the culture plate. Magnification ×100. **(C)** Total protein content of cultured HuCC-T1 cells (expressed as percentage of control cells protein) showing significant protein reduction upon rapamycin alone or rapamycin plus Q-VD-OPh administration. * *P* ≤ 0.01 ctrl vs. Rapa; ** *P* ≤ 0.005 ctrl vs. Q-VD-OPh +Rapa. **(D)** Representative Western blot experiment showing no effect of Q-VD-OPh caspase-10 inhibitor, rapamycin and their combination on cleaved PARP (apoptosis marker) in HuCC-T1 cells. β-Actin was used as loading control. Blot is representative of three independent experiments.

We tested on HuCC-T1 cells the effects of autophagy activation via Q-VD-OPh treatment and via rapamycin on the well-known proliferation marker PCNA (Mancino et al., 2009). Rapamycin is an autophagy inducer since it inhibits mTOR complex 1 (mTORC1) (Querfurth and Lee, 2021). Upon Q-VD-OPh alone, rapamycin alone or Q-VD-OPh combined with rapamycin administration, cells displayed reduction of PCNA level as compared to untreated cells in Western blot experiments (Figure 4A). We confirmed similar result in OZ cells, another iCCA cell line (Yoshikawa et al., 2009) (Supplementary Figure S2). The effect of Q-VD-OPh, rapamycin or their combined treatment in inducing PCNA decrease is associated with size reduction of the cell colonies as shown in the phase contrast images of HuCC-T1 cultures (Figure 4B). Colony size reduction also correlated with protein content reduction of lysed cell plate (Figure 4C). The decrease of colony size and protein content upon Q-VD-OPh, rapamycin or their combined treatment was not dependent on apoptosis upregulation since no difference regarding cleaved PARP-1 were observed upon the different treatments (Figure 4D). Similar colony size reduction upon Q-VD-OPh, rapamycin or their combined treatment was found in OZ cells (Supplementary Figure S3).

## 4 Discussion

Autophagy represents a key process controlling the biology of normal as well as pathologic conditions. The present work demonstrates autophagy impairment in iCCA (intrahepatic cholangiocarcinoma) cells which display lower LC-3II and higher p62 protein levels under basal culture conditions, as compared to healthy cholangiocytes (Figure 1). The high levels found in cell lysates *in vitro* were found to match a significant increase of p62 transcript levels in CCA biopsies as compared to normal healthy controls, according to expression levels reported in a public GEPIA2 (Figure 1). These findings parallel those obtained in glioma tissues, where p62 protein upregulation has been shown to be associated to p62 mRNA increase and autophagy impairment (Deng et al., 2019). We also show significant increase of p62 transcripts in melanoma and thymoma patients (Figure 1) confirming that p62 increase may represent a feature present in different solid and hematologic cancers. Remarkably, p62 is a diagnostic biomarker for early stage melanomas (Tang et al., 2016) and p62 is emerging as a possible therapeutic target in melanoma (Karras et al., 2019; Fei et al., 2022). Further, high p62 expression in lymphoma shows significant positive correlation with disease relapse (Kyriazopoulou et al., 2022). We have previously demonstrated high p62 expression in primary sclerosing cholangitis (PSC) representing a chronic inflammatory cholangiopathy frequently complicated by CCA (Carpino et al., 2019). Together with other groups we have also shown the important role of autophagy in promoting cholangiocyte differentiation and maintaining cholangiocyte homeostasis (Chen et al., 2018; Tomaipitinca et al., 2019; Casini et al., 2022).

The current study also highlights two possible mechanisms involved, namely, TFEB and caspase-10-c-FLIP enzymatic complex. TFEB is a transcription factor controlling cell fate in mammalian liver. TFEB has been previously shown highly expressed in biliary compartment as well as in progenitor/ductal cells while it is weakly expressed in mature hepatocytes (Pastore et al., 2020). Recent studies have elucidated the pivotal role of this transcription factor in promoting autophagy at transcriptional level by inducing different genes involved in autophagy flux and lysosomal biogenesis (Di Malta et al., 2019). Here we demonstrate different TFEB protein expression and intracellular localization in iCCA cells as compared to cholangiocytes. We found, in fact, very low TFEB protein expression and absent nuclear localization in iCCA cells, consistently with their impaired autophagy. Conversely, cholangiocytes display high TFEB protein expression with a localization both in cytoplasm and nucleus, consistently with their active autophagy. A doublet band found in total lysates from the control cells H69 might correspond to phosphorylated and unphosphorylated TFEB isoforms (Napolitano and Ballabio, 2016); interestingly, such doublet is not present in iCCA cells (Figure 2), consistently with their impaired autophagy.

We also addressed involvement of caspase-10 in controlling autophagy, as previously shown in multiple myeloma (Carroll and Martin, 2013). Caspase-10-c-FLIP enzymatic complex has been previously shown to inhibit autophagy by cleaving the BCL2-interacting protein BCLAF-1. This is a strong inducer of autophagy that acts by displacing beclin-1 from BCL2 in order to let beclin-1 promote autophagosome formation (Lamy et al., 2013). Under our experiments the autophagy deficiency observed in iCCA cells is consistent with higher c-FLIP andcaspase-10 levels as well as higher caspase-dependent cleaved BCLAF-1, as compared to cholangiocytes (Figure 3). Remarkably, we also show higher c-FLIP and caspase-10 transcripts inCCA biopsies as compared to controls (Figure 3). This result confirms our previous immunohistochemical results demonstrating high c-FLIP expression in iCCA specimens as compared to normal specimens (Carnevale et al., 2017).

We therefore concluded that BCLAF-1 may be responsible for autophagy impairment observed in iCCA cells through caspase-10-c-FLIP complex cleavage. Upon caspase-10 inhibitor supplementation autophagy is re-activated in iCCA cells as shown by LC3II increase (Supplementary Figure S1). We also show that rapamycin and caspase-10 inhibitor lead to PCNA decrease sand reduced colony size of treated cells (Figure 4). Such result was confirmed in an additional iCCA cell line (i.e., OZ cell line). These data, at least partially, confirm previous data showing that rapamycin, by inhibiting mTOR signalling, is able to reduce cell proliferation of both HuCCT1and OZ cells (Okada et al., 2009). Our data also support previous evidence that pterostilbene, a natural compound found in the extracts of blueberries, acts as an anti-cancer agent suppressing CCA cells proliferation via induction of autophagic flux (Wang et al., 2019). Previous studies have shown a pivotal role of the mTOR signalling cascade in primary liver cancer, including CCA, where upon hyper activation, mTOR signalling promotes cell proliferation contributing to tumour progression (Wu et al., 2019; Lu et al., 2021). We believe that iCCA cells autophagy impairment observed in the present work may be strictly dependent on mTOR signalling hyperactivation. An inverse relation between autophagy and mTOR complex 1 (mTORC1) has been previously shown. Specifically, mTORC1 suppresses autophagy via phosphorylation-dependent inhibition of ULK1/2 (Unc-51 like kinase) and the VPS34 (Kim and Guan, 2015).

Finally, the increase of p62 protein levels observed *in vitro* in HuCC-T1 cells was supported by the observation that p62 gene expression levels are also increased in cholangiocarcinoma patients (Figure 1). Only few other solid cancers share such feature, namely, two cancers strictly related to cholangiocarcinoma (namely, liver hepatocellular carcinoma and pancreatic cancers), melanoma and thymoma. We have previously highlighted the role several autophagy-related genes may have in melanoma (Scatozza et al., 2020; D’Arcangelo et al., 2018).

## 5 Conclusion

Our results demonstrate: i) autophagy impairment in iCCA cells, displaying lower LC-3II and higher p62 protein levels, as compared with healthy cholangiocytes; ii) significant modulation of transcripts levels of autophagy-related genes in CCA biopsies from CCA patients as compared to normal healthy controls on public databases; iii) two novel mechanisms controlling autophagy in this cancer model; iv) biological effects of autophagy induction in iCCA cells.

It is known that p62 plays a key role in autophagy and represents a multifunctional signalling hub by interacting with several pathways controlling inflammation and oxidative stress (Taniguchi et al., 2016). p62 positively regulates the transcription factor NF-κB, a central player in inflammation which promotes the development and proliferation of cancer cells (Taniguchi and Karin, 2018). Furthermore, accumulation of p62 activates Nrf2, a central regulator of stress resistance, particularly against ROS and oxidative stress (Shimizu et al., 2016). Remarkably, cancer cells show advantage from high Nrf2 level and increased ROS resistance (Saito et al., 2016). Therefore, our data may add useful information to develop anticancer strategies targeting autophagy or p62-regulated signalling pathways. We suggest that promoting p62 degradation via autophagy activation through caspase activity inhibition might represent a possible approach in counteracting growth of iCCA cells and might be considered in addition to mTOR inhibitors previously proposed as a promising approach (Koustas et al., 2021; Lu et al., 2021).

## Data Availability

The original contributions presented in the study are included in the article/Supplementary Materials, further inquiries can be directed to the corresponding authors.
