# Supplementary figures and images for: Autophagy impairment in human bile duct carcinoma cells
Source: Front Physiol. 2023 Sep 29;14:1249264. doi: 10.3389/fphys.2023.1249264 (PMC10570450; doi:10.3389/fphys.2023.1249264)

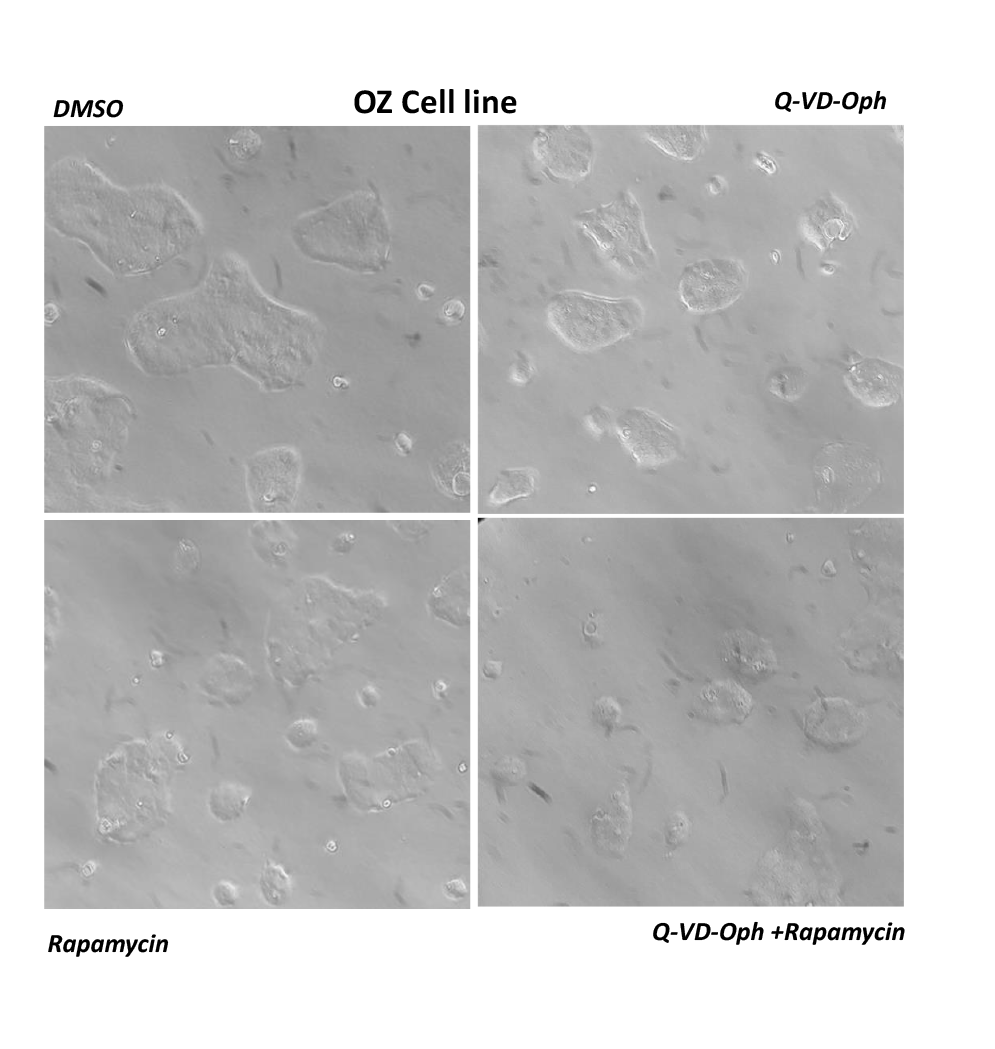

Supplement: Supplementary file 1 [file Image3.TIF]

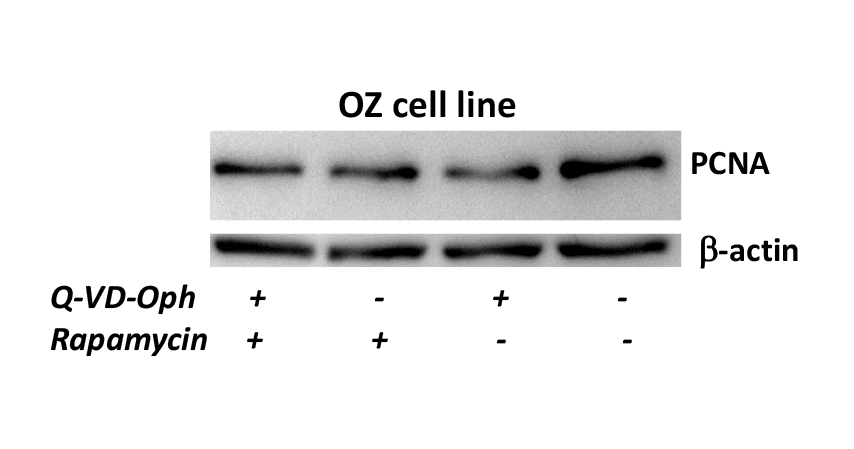

Supplement: Supplementary file 2 [file Image2.TIF]

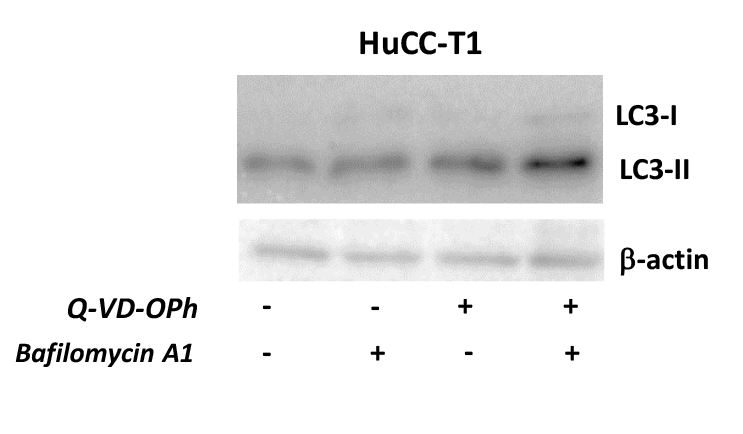

Supplement: Supplementary file 3 [file Image1.TIF]
